# Detrimental Effects of UVB on Retinal Pigment Epithelial Cells and Its Role in Age-Related Macular Degeneration

**DOI:** 10.1155/2020/1904178

**Published:** 2020-08-12

**Authors:** Camille Keisha Mahendra, Loh Teng Hern Tan, Priyia Pusparajah, Thet Thet Htar, Lay-Hong Chuah, Vannajan Sanghiran Lee, Liang Ee Low, Siah Ying Tang, Kok-Gan Chan, Bey Hing Goh

**Affiliations:** ^1^Biofunctional Molecule Exploratory Research Group, School of Pharmacy, Monash University Malaysia, 47500 Bandar Sunway, Selangor Darul Ehsan, Malaysia; ^2^Novel Bacteria and Drug Discovery Research Group, Microbiome and Bioresource Research Strength Jeffrey Cheah School of Medicine and Health Sciences, Monash University, 47500 Bandar Sunway, Malaysia; ^3^Medical Health and Translational Research Group, Jeffrey Cheah School of Medicine and Health Sciences, Monash University Malaysia, 47500 Bandar Sunway, Selangor Darul Ehsan, Malaysia; ^4^Advanced Engineering Platform, Monash University Malaysia, 47500 Bandar Sunway, Selangor, Malaysia; ^5^Department of Chemistry, Drug Design and Development Research Group (DDDRG), Centre for Theoretical and Computational Physics, Faculty of Science, University of Malaya, 50603 Kuala Lumpur, Malaysia; ^6^Institute of Pharmaceutics, College of Pharmaceutical Sciences, Zhejiang University, 866 Yuhangtang Road, Hangzhou 310058, China; ^7^Key Laboratory of Biomedical Engineering of the Ministry of Education, College of Biomedical Engineering & Instrument Science, Zhejiang University, Hangzhou 310058, China; ^8^Chemical Engineering Discipline, School of Engineering, Monash University Malaysia, Jalan Lagoon Selatan, Bandar Sunway, 47500 Subang Jaya, Selangor, Malaysia; ^9^Division of Genetics and Molecular Biology, Faculty of Science, Institute of Biological Sciences, University of Malaya, Kuala Lumpur, Malaysia; ^10^International Genome Centre, Jiangsu University, Zhenjiang, China; ^11^College of Pharmaceutical Sciences, Zhejiang University, 866 Yuhangtang Road, Hangzhou 310058, China; ^12^Health and Well-Being Cluster, Global Asia in the 21st Century (GA21) Platform, Monash University Malaysia, Bandar Sunway 47500, Malaysia

## Abstract

Retinal pigment epithelial (RPE) cells are an essential part of the human eye because they not only mediate and control the transfer of fluids and solutes but also protect the retina against photooxidative damage and renew photoreceptor cells through phagocytosis. However, their function necessitates cumulative exposure to the sun resulting in UV damage, which may lead to the development of age-related macular degeneration (AMD). Several studies have shown that UVB induces direct DNA damage and oxidative stress in RPE cells by increasing ROS and dysregulating endogenous antioxidants. Activation of different signaling pathways connected to inflammation, cell cycle arrest, and intrinsic apoptosis was reported as well. Besides that, essential functions like phagocytosis, osmoregulation, and water permeability of RPE cells were also affected. Although the melanin within RPE cells can act as a photoprotectant, this photoprotection decreases with age. Nevertheless, the changes in lens epithelium-derived growth factor (LEDGF) and autophagic activity or application of bioactive compounds from natural products can reverse the detrimental effect of UVB. Additionally, *in vivo* studies on the whole retina demonstrated that UVB irradiation induces gene and protein level dysregulation, indicating cellular stress and aberrations in the chromosome level. Morphological changes like retinal depigmentation and drusen formation were noted as well which is similar to the etiology of AMD, suggesting the connection of UVB damage with AMD. Therefore, future studies, which include mechanism studies via *in vitro* or *in vivo* and other potential bioactive compounds, should be pursued for a better understanding of the involvement of UVB in AMD.

## 1. Introduction

The human eye is a complex structure that allows us to see the world in its many colors, shades, and shapes. This requires light from the environment to enter the eye and be focused on the retina which contains photoreceptors. The human eye mainly consists of three distinct layers: (1) the outer region, made up of the cornea and sclera; (2) the middle layer, consisting of the iris, choroid, and ciliary body; and finally (3) the inner layer which is the retina. These three ocular layers surround three other transparent structures known as the aqueous humor, vitreous humor, and the lens [1]. As light enters the eye, it first passes through the conjunctiva, which is the transparent mucous membrane that covers the visible part of the sclera. It then passes through the cornea and aqueous humor and enters the vitreous humor via the pupil. The light rays then come into contact with the innermost layer of the eye—the retina [1].

**Figure 1:**
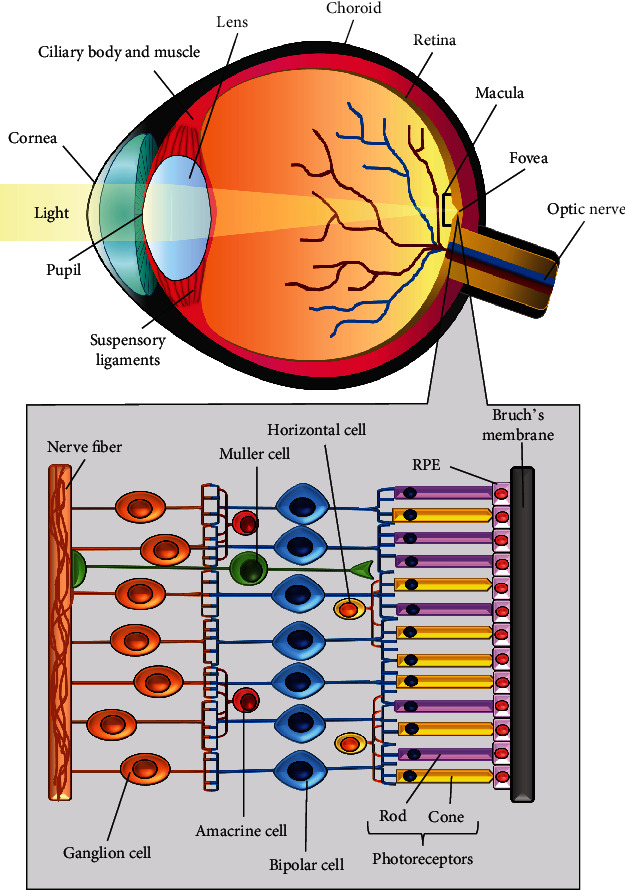
An illustrated anatomy of the eye and the cells present within the retina.

A key role of the retina is to convert light stimuli to neural impulses, which are then transmitted through the optic nerve to the occipital lobe [2]. The retina is made of the retinal pigment epithelium (RPE), Bruch's membrane, and a sensorineural layer which is also known as the sensory retina. Within this sensory retina are photoreceptors that convert the light signal to action potentials that are sent to the brain for processing [1]. Within the center of the retina lies the macula and in its center is the fovea. The fovea is a specialized region of the eye that has the highest visual acuity due to the absence of rods and a maximum cone density. The center of the fovea, which is the fovea pit, is also thinner as a result of the outward displacements of inner retinal neurons [3]. RPE is also crucial to the normal functioning of the retina due to its support towards the photoreceptors. RPE cells are hexanocuboidal neuroectodermal origin monolayer cells located in-between Bruch's membrane and the photoreceptor cells [4, 5]. The apical side of the RPE membrane faces the photoreceptor's outer segments of rods and cones, while the basolateral side faces Bruch's membrane, forming the blood-retinal barrier [6]. Key features of the RPE cells are their tight junctions between neighboring cells which functions to tightly control the transportation of fluids and solutes across the blood-retinal barrier. It also prevents plasma components and toxic molecules from entering the retina. The main function of the RPE is to (1) maintain the blood-retinal barrier (BRB) between the choroidal blood circulation and photoreceptors; (2) transport nutrients, ions, and water; (3) protect the outer retina against photooxidative damage by absorbing light; (4) perform phagocytosis and degrade detached distal portions of photoreceptors; (5) reisomerize all-*trans*-retinal to 11-*cis*-retinal; and finally (6) act as a vascular and neural protector by secreting cytokines and growth factors [7].  Figure 1 depicts an illustrated anatomy of the eye and the arrangements of cells within the retina.

The progressive deterioration of the macula in age-related macular degeneration (AMD) is the main cause of vision loss and blindness among the elderly, especially those above the age of 60 [8]. Early and intermediate stages of AMD are based on the extent and size of AMD-related retinal modifications. However, advanced stages of AMD are classified by either an irreversible loss of photoreceptors or the development of macular neovascularization [8]. There are two classifications of AMD, which is wet or dry AMD. Wet AMD is characterized by the loss of vision through subchoroidal neovascularization. It is through the growth of angiogenic blood vessels that penetrates through Bruch's membrane and the RPE layer into the subretinal space [9, 10]. This leads to exudation and bleeding which ultimately destroys the photoreceptors [9]. On the other hand, dry AMD which is more common among patients (~90%) occurs through the slow death of the RPE, choriocapillaris, and neuroretina, which then develops to the permanent loss of vision [9, 10]. While all eyes experience degenerative changes with aging, not all elderly develop the changes of AMD. The etiology of AMD appears to be multifactorial too. Daily exposure of our retina to oxygen and light; prolonged inflammation by harmful agents; and lifestyle choices like smoking, drinking alcohol, and our diet could contribute to mutations in nuclear and mitochondrial DNA. All these factors were suggested to be contributors to the pathogenesis of AMD [2].

As ultraviolet rays (UVR) from the sun can induce oxidative stress, it is one of the factors that have been known to cause damage to the eyes [11]. The sun produces three kinds of ultraviolet rays (UVR): UVA, UVB, and UVC. UVA has the longest wavelength, ranging from 320 and 400 nm, and a penetration level of 95% through the ozone; UVB has a penetration level of 5% and a range of 290-320 nm; and finally UVC with a range of 200-290 nm which is absorbed by our stratosphere [12]. The penetration level of UVA is constant throughout the day while UVB's penetration level will increase to its highest value of 5% at midday [13]. As our eyes take in light, they are also exposed to UVR which may cause damage to ocular tissues, but the human eye has unique UVR-filtering systems. UVR that is below 295 nm, which consists of some UVB, will be filtered by the cornea, especially at the anterior section of the cornea [14]. After passing through the cornea, the lens then proceeds to absorb most of the UVA and UVB wavelengths before they reach the retina [15]. Despite these protective barriers, a small portion of UV still reaches the retina, especially in children below the age of 10. However, as a person ages, the transmission of UVR decreases due to the increase of UV-absorbing chromophores within the lens [16]. Yet, this is not true for those who had undergone cataract surgeries who are again vulnerable to the harmful effects of UVR if the intraocular lens that replaces their lens is unable to filter these rays as effectively [17]. Those who take photosensitizing drugs are also at increased risk of phototoxicity towards the RPE [18, 19]. Besides that, places of higher altitudes were reported to increase by 30% in UVA and UVB exposure for every 1000 m. For example, a person hiking Mount Everest would be exposed up to 133% more UVR in comparison to those at the summit of Mount Whitney. Snow too reflects about 80% of UVR, increasing further the detrimental effects of UVR [20]. Finally, the depletion of the ozone layer contributes as well to greater fluxes of UVB on the earth's surface, which may contribute to the increase of UVB-induced age-related macular degeneration [21].

**Figure 2:**
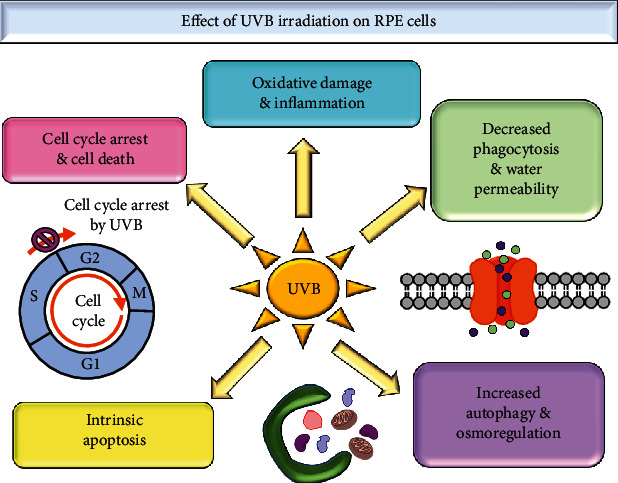
A summarized view regarding the modifications and damage inflicted on RPE cells when irradiated with UVB.

Hence, in this review, we look into the damage caused by UVB on RPE cells in both *in vitro* and *in vivo* models. From the studies that had been done, UVB irradiation induces not only oxidative damage but also inflammation and cell apoptosis via various pathways. The autophagy, phagocytosis, and water permeability of RPE cells are also altered following exposure to UVB. As damage to RPE could lead to macular degeneration, understanding the mechanism and pathways induced by UVB could aid in finding a treatment. On the other hand, natural products, which have proved to be a vast source of bioactive compounds, may be viable treatments against the detrimental effects of UVB.  Figure 2 shows a summary of the effects of UVB on RPE cells.

## 2. RPE Cell Lines Utilized in UVB Studies

Although the establishment of RPE cells is fraught with many challenges, *in vitro* studies on RPE cells have always been the better option for experimentation. This is due to the fact that *in vivo* studies on RPE are difficult to conduct because of its location, inability to regenerate, and the inhibition of drug transfusion across the blood-retinal barrier into the vitreous humor [22]. As with the establishment of any cell line, the goal was to obtain cell lines that were as similar to an *in vivo* model as possible and yet be able to continue to grow and expand *in vitro* without losing or gaining features over time. Several features of a good RPE cell line includes (1) similar morphology as *in vivo* RPE cells; (2) ability to form apical-basal polarization; (3) able to form zonula occludens or tight junctions with each other; (4) has polarized distribution of the ion transport system and perform fluid transport; (5) secretes extracellular matrixes such as collagen (types I, III, IV, and V), fibronectin, heparan sulfate proteoglycan, and laminin; (6) produces melanosomes; (7) forms intermediate filaments; (8) has organized actin and cytoskeletal elements; (9) develops asymmetric distribution of sodium pumps; (10) has phagocytotic ability; and (11) ability to metabolize vitamin A [23].

With those features in mind, various RPE cell lines like ARPE-19, D407, and DrRPE were raised.  Table 1 depicts some of the cell lines that were used in the investigation of the detrimental effects of UVB. These cell lines were either established previously or are primary cell lines that were obtained from animal models. Other primary human cell lines, obtained personally from different donors too, were used as well in some of the studies; however, their morphologies were either not studied or not mentioned in detail to be discussed in this review. Hence, they are not included in Table 1. Among the cell lines, both ARPE-19 and D407 are established human RPE cell lines by Dunn et al. [24] and Davis et al. [25], respectively. Each cell line has its characteristics and drawbacks such as the changes to the cell morphology and decrease in transepithelial resistance (TER) measurement, indicating an impairment in the cell barrier functions due to weakening cell polarization and tight junctions [22]. Yet, based on the different cell line characteristics, although ARPE-19 has varied pigmentation across the different cells, it is a much preferable cell line to study UVB damage. This is because it is essential that the RPE cells have pigmentation, mimicking actual RPE cells within the eyes, as melanin plays photoprotective roles within RPE cells [26]. Besides that, other studies had shown that with appropriate culture and differentiation of the cells, the ARPE-19 cells were able to better retain their phenotype and gene expressions [27, 28].

On the other hand, using primary cell lines does come with its advantages. One key advantage in utilizing primary RPE cell culture is the freedom to choose the human samples that best fit with the study of interest. For example, in the research done by Yacout et al. [29], RPE cells from a 78-year-old donor and a 67-year-old donor with dry AMD were used as the samples to better understand the changes in melanin within the cells as compared to younger RPE cells. Besides that, researchers too can utilize primary RPE cells from transgenic animal models to study genetic or age-related degenerative disorders as human donor cells are hard to obtain [30]. Nevertheless, despite primary RPE cell lines having an advantage of being closer in similarity to *in vivo* models, it is difficult to maintain its pigmentation and high TER during the expansion of the cells as described by Fernandez-Godino et al. [30] when cultivating mouse RPE. A similar loss of melanin granules too was reported by Eves et al. [31] when culturing primary human RPE cells beyond passages 3 and 4. Besides that, primary RPE cell lines too pose problems such as heterogeneity within the cultures, donor variability, short lifespan, slower growth, and relatively smaller amount of cells that can be obtained for experiments [32].

Other than human cell lines, a new animal model RPE cell line, known as DrRPE (*Danio rerio* RPE), had been developed by Nambi et al. [33]. These are RPE cells established from the globes of zebrafish. The established cells displayed epithelial-like morphology, had diploid chromosomes, and has the ability to produce retisomes which are essential storages to replenish the visual chromophore, 11-*cis*-retinal, ensuring proper visual function even during low dietary vitamin A intake [33, 34]. Although the cells slowly lost their pigmentation over time, they were still able to be passaged for more than 75 times for 2 years and showed a high plating efficiency, displaying its stability as an established cell line. Besides that, it had also been reported to have similar functional properties of RPE cells *in vivo*, had all the potential characteristics of mammalian RPE cells, and is cost-effective and easy to maintain [33]. Other species such as rabbits, amphibians, cows, mice, and rats too are good sources to obtain RPE cells as they are much easily available as compared to human eyes [35–39].

In short, *in vitro* cell lines do provide certain benefits in the study of RPE mechanism and various ocular pathologies as compared to *in vivo* models. Yet, despite having many choices of RPE cell lines, many researchers have turned to used ARPE-19 as their model of investigation. According to our point of view, this is probably due to the commercial availability of the cell line and the elimination of any need for ethics application, be it human or animal. Besides that, as this cell line is widely used in many types of RPE research, it is most likely better characterized, and thus, comparisons and conclusions of results can be drawn much quicker with the utilization of this cell line. Finally, the ARPE-19 cell line would be more stable across passages as compared to primary cell cultures. However, researchers should always be encouraged to choose a cell line that would best fit with their study of interest.

## 3. UVB Induced Dysregulation on *In Vitro* RPE Cells

### 3.1. Direct DNA and the Induction of Oxidative Stress in RPE Cells

Although there is still an ongoing debate around the penetrance level of UVB that reaches the RPE layer, it is inarguable that even a small percentage of UVB penetrance can be very detrimental in the long term. When bovine RPE was irradiated by 0.09 J/cm^2^ of UVB, DNA damage was detected via the formation of the tail in comet assay as compared to the control. The unirradiated cells still displayed a tightly packed DNA within the nucleus [44]. A similar effect was also seen in ARPE-19 cells that were irradiated with UVB. The cells were seen to decrease in cell viability in proportion with the increase in the energy level [45]. This increase in cell death and DNA damage after irradiation is due to the ability of UVB in forming cyclobutane pyrimidine primers and pyrimidine-pyrimidone (6-4) photoproducts [46]. A study conducted in 1999 evaluated the ability of RPE cells in repairing DNA damage [47]. RPE cells were firstly separately irradiated with either 0-0.09 J/cm^2^ UVB or 0-0.9 J/cm^2^ UVA. After that, the DNA damage was evaluated using comet assay either immediately after irradiation, an hour, or 24 hours after the UV exposure. The results indicated that UVA was much weaker than UVB in inducing DNA damage and cell death, but after exceeding the tolerable amount of DNA damage, RPE cells opt to initiate cell apoptosis instead of repairing the DNA damage [47]. This shows the detrimental damage of UVB towards cells that were exposed to it.

Besides inducing DNA damage, UVB irradiation too was reported to increase reactive oxygen species (ROS) levels initiating oxidative stress in RPE cells. The retina is located in an environment that is rich in oxygen, which makes it a prime area in the generation of ROS [48]. The RPE and photoreceptors are also constantly and consistently exposed to light, making them particularly susceptible to oxidative damage [49]. The pathophysiology of AMD is a progressive one, but it has been shown that among the core cell biology concepts related to its development in AMD and aging, oxidative stress induces RPE injury and thus results in chronic inflammation within the choroid and Bruch's membrane. This is then followed by, possibly, injury and inflammation in the choriocapillaris, leading to abnormal extracellular matrix formation, which could alter the diffusion of the nutrients to the RPE and retina. This then comes back as a full circle to further damage and ultimately leads to the atrophy of the retina, RPE cells, choriocapillaris, etc. [50].

**Figure 3:**
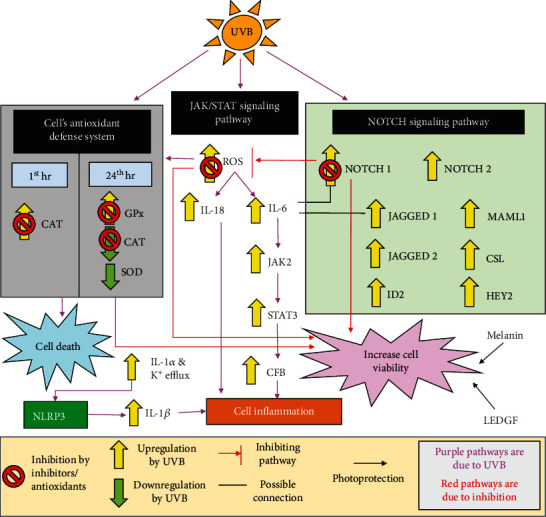
UVB irradiation activates the RPE cell's antioxidant defense system and two signaling pathways, JAK/STAT and NOTCH signaling pathways. Melanin and LEDGF too act as a photoprotection against UVB. Inhibition of different protein expressions or the formation of ROS negates the damaging effect of UVB and increases cell viability.

According to Cao et al. [51], ROS levels were seen to be elevated as early as 30 minutes postirradiation. Other than the increase in ROS, irradiated ARPE-19 cells also exhibited a significant increase in catalase (CAT) within the first hour of exposure, while the other enzymes such as glutathione peroxidase (GPx) and lipid peroxidation increased 24 hours after irradiation. This is coupled with a slight reduction in superoxide dismutase (SOD) activity as well as a significant decrease in catalase activity. Although there had been a spike in the enzymes in response to UVB irradiation, there is still a significant amount of cell death, suggesting that the antioxidant system may be overwhelmed by the damage caused by UVB [52]. However, when treated with antioxidant trolox and/or lutein, the catalase activity showed an opposite change in activity whereby it decreased and increased in activity at the 1^st^ hour and 24^th^ hour after irradiation, respectively. Cell viability of ARPE-19 cells was also shown to have increased. Activity levels of other antioxidants like GPx also decreased at 24 hours after treatment with trolox and lutein [52]. This suggests that with the addition of trolox and/or lutein, the cells were able to overcome the UVB damage by suppressing the spike in ROS and thus “rescuing” the cells from cell death as depicted in Figure 3. The changes in the antioxidant activity too supported the indication that trolox and/or lutein were able to reduce the damage caused by UVB. An interesting point to note is the changes in the catalase activity in the 1^st^ hour and 24^th^ hour in response to UVB irradiation and its complete reversal when the cells were treated with trolox and/or lutein. Previously, a study on keratinocytes had reported that catalase itself produces oxidants in response to UVB irradiation while the increased GPx aids in diminishing the catalase-induced ROS [53]. Other studies also had shown that overexpression of catalase at 16 h onwards after UVB exposure was able to negate the increase of UVB-induced ROS in normal and diseased keratinocyte cells [54, 55]. Whether changes in catalase activity could affect the cell viability in RPE is still unknown; however, it does warrant more studies especially if there is a possibility that it could contribute to RPE degeneration-related diseases including AMD.

### 3.2. Photoprotection of Melanin and LEDGF against UVB

Melanin had been suggested to function as an antioxidant due to the ability to scavenge free radicals [56]. Melanin also binds to metal ions, such as Fe(II), Cu(II), Ca(II), and Zn (II), and sequester them, thus inhibiting these metal ions from being reduced by cellular components and inducing oxidative stress [57]. Other research had also shown that melanin can protect RPE from the oxidation of A2E by inhibiting them [58]. However, this inhibitory effect dramatically decreases while lipofuscin increases in aged human samples [58, 59]. Lipofuscin granules are the result of incomplete degradation of phagocytosed material that has autofluorescence properties from its hydrophobic component, A2E; they cause further damage to the RPE cells in various ways including reducing the phagocytic capacity of RPE, lipid peroxidation, membrane blebbing, and cytoplasmic vacuolation [59–63]. It had also been reported to inhibit lysosomal function and further reduce the antioxidant capacity of RPE cells by photogeneration of even more ROS, which increases the oxidative stress within the cells [64]. Although low levels of A2E had been reported to be able to protect RPE cells from UVB-induced DNA damage by acting either as an inner filter to absorb photons or perhaps a ROS quencher, high accumulation of A2E induces cell apoptosis instead [65, 66].

Recently, a study done by Yacout et al. [29] displayed that there is a significant loss of melanosomes in a 67-year-old AMD donor's RPE cells as compared to a 78-year-old healthy donor's RPE cells. However, both eyes are relatively lower in melanosome as compared to those from a 19-year-old donor's RPE cells. Further measurement using MALDI-TOF showed that the melanin in the younger donor RPE has a higher *m*/*z* range as compared to the older but healthy donor RPE, which suggests that there is a loss of melanin as one aged [29]. This data that was obtained is also supported by other studies that displayed a downward trend in melanin content within the RPE as one grows older [26, 67]. The decrease is even calculated to be nearly 2.5-fold difference between 10 and 90 years of age [26]. Additionally, when calf melanosomes were oxidatively degraded with peroxide, the *m*/*z* distribution obtained was similar to the *m*/*z* of those obtained from the AMD donor, signifying that AMD patients may be experiencing extensive degradation of melanin in their RPE cells [29]. To look further into the photoprotective properties of melanin, some studies had proceeded to introduce artificial repigmentation with synthetic melanin or melanin obtained from other donors' eyes. This is because it is very common that *in vitro* RPE cells lose their melanin pigments over time and that melanogenesis cannot be reactivated in the cultured cells via exposure to UVR [29, 31, 43]. One study compared the difference in the photoprotective properties of melanin from young and old RPE cells against UVB. ARPE-19 cells were firstly allowed to uptake melanin from 6 months and younger calf or melanin obtained from 72- and 74-year-old healthy donor eyes. Then, the cells were irradiated with UVB before the levels of ROS and nitric oxide (NO) were measured in the cells. After irradiation, it was noted that there was a significant 43% decrease in ROS levels in cells containing calf melanin, while cells containing the melanin from old donor eyes displayed a 28% decrease in ROS, as compared to the unpigmented RPE cells. On the other hand, cells with calf melanin experienced a decrease in NO levels after irradiation. However, the opposite was seen for cells with melanin from the old donor eyes, where the level of NO was seen to be elevated after UVB [29].

From the data obtained, two observations can be made. (1) As a person ages, the melanin content in RPE cells decreases, and this decrease is even more evident in AMD patients. (2) Although melanin does have photoprotective properties by attenuating the oxidative stress generated through UVB, this ability decreases and even becomes prooxidant in later stages of life. One factor that was speculated to contribute to the decrease in melanin RPE with age is the inability of melanin in RPE to renew, unlike keratinocyte cells. Rather, the synthesis of melanin from L-DOPA in RPE occurs during fetal life, but this ability is later lost in adult RPE cells due to the lack of premelanosomes and tyrosinase activity [68]. However, this assumption on RPE melanogenesis is still being challenged as there are hints that RPE undertakes a different melanogenesis mechanism as compared to keratinocytes [69]. Nonetheless, the visible reduction of melanin in the RPE cells in aged and AMD patients is irrefutable. According to other studies, the loss of melanin and melanin photoprotection over time could also be due to the formation and accumulation of both lipofuscin and melanolipofuscin and photodegradation of melanin. Melanolipofuscins are granules that were reported to be made of a dark inner core, with compositions similar to melanosomes, and a brighter outer shell, similar to the composition of lipofuscin, which encapsulates it [70]. They are also accumulated over time and were suggested to be the cause of the time-dependent decrease in the number of melanosomes found in RPE [71]. The accumulation of melanolipofuscin is also phototoxic towards RPE cells as confirmed by Warburton et al. [72] RPE cells containing melanolipofuscin revealed a decrease in cell viability by 58% after blue light irradiation. As for the role of lipofuscin in melanosome degradation, it was found that lipofuscin is able to generate superoxide anions under light irradiation [71, 73]. To further understand the mechanism of melanosome destruction, a study was done whereby human RPE melanosomes were treated with potassium superoxide or irradiated with blue light to mimic the conditions within the RPE. As expected, superoxide anions either from potassium superoxide or from blue light exposure induced degradation of melanosomes and production of fluorescent products. The number of melanosomes also reduced dose-dependently after the treatment, demonstrating the decrease of melanosomes with time in RPE [71].

Besides the damaging effect of melanolipofuscin, the photodegradation of melanin is another concern. To better understand the photodegradation of melanin, a study was done by irradiating synthetic DOPA-melanin (a combination of DOPA and melanosomes obtained from bovine RPE) with UVR up to 52 h [74]. The results displayed that long-term radiation leads to not only the destruction of DOPA-melanin and production of fluorescent products but also a decrease in antiradical activity and concentration of dark- and light-induced paramagnetic centers of DOPA-melanin [74]. Although such high levels of radiation on RPE may not be possible naturally, Dontsov et al. [74] suggest that UVR may still play a role in melanin photodamage via oxidative stress. This is because oxidative damage by hydrogen peroxide showed that DOPA-melanin was quickly destroyed and produced fluorescent products in the presence of oxidant [74]. Another study too demonstrated reduced electron density and melanin in porcine melanosomes after exposing them to visible light up to 48 hours. There are also prominent changes to the morphology of the melanosomes where the surface of the melanosomes becomes rougher, misshapen, and fragmented. Finally, the ability of melanin to bind to metal ion was seen to decrease by 30-50% and 60-80% after 24 and 48 h of light exposure, respectively. This indicates the decrease of melanin to function as an antioxidant over prolonged exposure to light [75]. Hence, in summary, although melanin does provide photoprotection to the RPE cells against UVB, this photoprotection will decrease over time and potentially contribute to the pathophysiology of AMD.

On the other hand, another way to negate the degenerative effect of UVB on RPE cells is by increasing the expression of LEDGF. LEDGF is a member of the hepatoma-derived growth factor protein family and is upregulated under oxidative and heat stress. The increase in LEDGF expression was found to activate the expression of stress-related genes, like Hsp27 and *α*B-crystallin, by binding to heat shock element (HSE) and stress regulatory element (STRE). Thus, the elevation of LEDGF protects the cells against environmental stresses [76]. Previously, the potential of LEDGF as a protective agent towards the retina had been investigated by Machida et al. [77]. In their experiment, three different types of rats with different ocular damage were injected with LEDGF fused with glutathione-S-transferase as part of their treatment. The results indicated that in two of the models, normal rats that were induced to have light damage and Royal College of Surgeons (RCS) rats that had inherited photoreceptor degeneration had significant preservation of the cones and rod photoreceptors. However, the preservation of the photoreceptor by LEDGF was not seen in the transgenic rat model with rhodopsin mutation Pro23His (human retinitis pigmentosa model), suggesting that LEDGF has the potential to reverse or slow retinal damages. The potential of LEDGF against UVB-induced RPE cell damage was seen when primary human RPE was treated with external LEDGF-heparin for 2 hours before UVB exposure. The yield portrayed a significant increase in the number of live cells at 24 and 72 hours after irradiation as compared to the control cells treated with heparin alone. When investigated further, LEDGF-heparin treatment was discovered to attenuate the detrimental effect induced by UVB by reducing the DNA strand breaks in RPE cells. A similar effect was also seen when the RPE cells were treated with hydrogen peroxide instead to mimic cells under oxidative stress. The upregulation of heat shock protein (Hsp)27 too was recorded in cells that were treated with LEDGF-heparin. However, Hsp90 and *α*B-crystallin were not upregulated. Thus, it can be said that LEDGF is capable of being a photoprotectant to RPE cells by attenuating the oxidative damage induced by UVB radiation.

### 3.3. Instigation of NLRP3 Inflammasome in UVB-Stressed RPE

As oxidative damage and inflammation come hand in hand in AMD, the impact of UVB on the inflammatory pathway had been studied as well. One particular inflammatory pathway that is implicated is the activation of inflammasomes due to UVB-induced oxidative stress. The inflammasome is a multicomplex protein that activates the inflammatory caspase, initiating the processing and secretion of proinflammatory cytokines [78]. It is a large intracellular signaling platform containing a cytosolic pattern recognition receptor with either an absent in melanoma 2- (AIM2-) like receptor or a nucleotide-binding oligomerization domain-like receptor (NLR) [79]. Among many inflammasome complexes, one that is widely characterized is the nucleotide-binding domain and leucine-rich repeat pyrin containing protein 3 (NLRP3) inflammasome. The role of this inflammasome was suggested to control the maturation of two proinflammatory cytokines, interleukin- (IL-) 1*β* and IL-18. After being activated, NLRP3 then forms a multiprotein complex with adapter apoptosis-associated speck-like protein containing a C-terminal caspase recruitment domain (ASC) with pro-caspase-1, which is then cleaved to form the activated form of caspase-1 [79]. The activated caspase-1 then proceeds to convert pro-IL-1*β* and pro-IL-18 into their mature forms [80]. There are three known mechanisms that can trigger the activation of the NLRP3 inflammasome: (1) the generation of ROS, (2) lysosomal destabilization, and (3) the efflux of potassium (K^+^) [81–83].

Some studies had proven that NLRP3 plays an important role in the pathophysiology of AMD. When the mRNA of macular lesions was isolated from both wet and dry AMD patient eyes, a significant upregulation of NLRP3 pro-IL-1*β* mRNA was reported in both types of AMD in comparison to normal controls [84]. To confirm the activation of NLRP3 in the retina of AMD patients, Wang et al. [84] proceed to induce oxidative stress and inflammation on ARPE-19 cells. The results yielded an upregulation of NLRP3, IL-1*β*, IL-18, and cleaved caspase-1 in both mRNA and protein expression [84]. Besides that, plasma membrane pores were also seen to form occasionally in the stressed cells, suggesting the induction of pyroptosis, a proinflammatory form of cell death, by NLRP3 activation [78, 84]. A similar increase in NLRP3, IL-1*β*, and IL-18 in ARPE-19 cells too was echoed in another study which had induced oxidative stress and lipid peroxidation with lipopolysaccharide (LPS) and 4-hydroxy-2-nonenal (HNE). In their research, the increase in IL-1*β* and IL-18 was suggested to be caused by the increase of pro-IL-1*β* and pro-IL-18 by LPS, which was then converted to the mature form by HNE via the NLRP3 inflammasome pathway [85]. Wang et al. [84] too demonstrated that the upregulation of IL-18 and NLRP3 was observed as well in their *in vivo* studies on mice. The increase of both transcripts was detected in the retina of DKO *rd8* mice, which were genetically modified to mimic the pathology of AMD. Additionally, the expression was observed to increase with age [84]. Based on the studies that were conducted, it is clear that the NLRP3 inflammasome pathway is an important component in the study of AMD.

As UVB also induces oxidative stress in RPE cells upon irradiation, perhaps the activation of NLRP3 inflammasome signaling may occur as well in RPE cells. To verify this, IL-1*α* primed and unprimed ARPE-19 cells were irradiated with 2.29 J/cm^2^ UVB by Korhonen et al. [86]. In both samples, ROS levels were elevated and significant expression of IL-18 into the surrounding media was detected as well. However, interestingly, only the primed cells positively expressed IL-1*β* while the unprimed cells did not show any increase in IL-1*β* protein expression. Furthermore, when the expression of cleaved caspase-1 was analyzed, it was noted that the expression of the protein was almost doubled in primed cells as compared to the unprimed cells. Nevertheless, both samples did indeed display a significant upregulation in cleaved caspase-1 as compared to the unexposed control. After that, to further investigate the involvement of NLRP3 inflammasome in UVB-induced RPE damage, the NLRP3 inflammasome was silenced in the primed samples. Once again, interestingly, this silencing of NLRP3 significantly reduced the expression of IL-1*β* but did not affect the expression of IL-18. Additional mechanism studies either through suppression of intracellular ROS or the inhibition of K^+^ efflux further revealed that in UVB-induced RPE cell damage, the expression of IL-18 is regulated by the presence of ROS while IL-1*β* expression is controlled by NLRP3 inflammasome which is activated by K^+^ efflux as depicted in Figure 3 [86].

Based on this data, it can be seen that, firstly, the expression of IL-18 is independent of the NLRP3 inflammasome pathway in UVB-damaged RPE cells. Secondly, the induction of expression of IL-*β* in UVB-damaged RPE cells requires extracellular IL-1*α* priming. One explanation for this independent expression of IL-18 from NLRP3 could be the activation of other noncanonical pathways such as the caspase-8 activation pathway. Bossaller et al. [87] described that in bone marrow-derived macrophages, it is possible to induce the maturation of IL-1*β* and IL-18 without the involvement of inflammasome or RIP3. This is achieved through the activation of Fas, which in turn recruits and activates caspase-8 through FADD, bringing about the expression of matured IL-1*β* and IL-18. On the other hand, another study on intestinal epithelial cells suggests that caspase-8 may act as an alternative route for IL-18 maturation through NLRC4 inflammasome when caspase-1 was inhibited [88]. Despite these speculations, more research still needs to be done to fully understand the mechanism and effect of UVB exposure on RPE. Besides that, i*n vivo* research that involves the whole retina too needs to be conducted as there is a potential that the activation of the inflammasome pathway in RPE requires prompting by surrounding cell conditions. For example, the formation of drusen in AMD patient eyes was found to initiate the activation of the NLRP3 inflammasome pathway, but the elevated NLRP3-mediated IL-18 may have a protective role on the retina through its inhibition on VEGF [89]. As these factors come into play, changes in RPE cell expression will certainly occur as well.

### 3.4. Activation of the NOTCH and JAK/STAT Signaling Pathways in RPE Cells

The NOTCH signaling pathway is a highly conserved pathway that can influence apoptosis, proliferation, and cell fate [90], though its role in different cells appears to have a high degree of variability. In keratinocytes, the role of NOTCH was seen to be very different for different cell lines when exposed to UVB. As reported by Mandinova et al. [91], the activation of the NOTCH 1 signaling pathway had a prosurvival effect against UVB via downregulation of FoxO3a, a key apoptotic gene, in primary keratinocyte cell culture. On the other hand, no changes in expression of NOTCH 1 and its ligand, JAGGED 1, were seen after UVB irradiation of HaCaT (normal) and SCL-1 (malignant) human keratinocytes, even when subjected to treatment with vitamin D [92]. In RPE, NOTCH signaling has been suggested to be involved in the patterning of the eye, epithelial-mesenchymal transition, cell proliferation, and cell migration [93–96]. Research on wet AMD had shown that the activation of the canonical NOTCH signaling pathway via JAGGED 1 induction was able to decrease the volume of choroidal neovascularization lesions of wet AMD by 4-fold in rats. While the inhibition of the NOTCH pathway exacerbated the condition of the lesions, showing that the NOTCH signaling pathway is able to negatively affect ocular angiogenesis [97]. Seeing that changes in the NOTCH signaling pathway could affect severity AMD, it is essential to study the effect of UVB on the NOTCH signaling pathway in RPE cells.

In an experiment, ARPE-19 cells experienced an increase in NOTCH 1 and 2 mRNA expressions after irradiation at 25 and 50 mJ/cm^2^ UVB. The gene expressions of other components of NOTCH signaling pathways, which includes NOTCH ligands (JAGGED 1 and JAGGED 2), transcriptional coactivators (MAML1), transcription factor (CSL), and target genes (HEY2 and ID2), were upregulated as well. To determine if the NOTCH pathway was involved in the survival of RPE cells, NOTCH 1 and 2 were silenced using shRNA while the cells were irradiated with UVB. Silencing of NOTCH 1 did not change the ROS levels and the apoptotic cell number, but silencing of NOTCH 2 not only decreased ROS levels but also decreased apoptosis cell numbers and increased cell viability [98]. This suggests that the NOTCH pathway, specifically NOTCH 2, is involved in UVB-induced RPE cell apoptosis.

Besides that, the multiple roles of NOTCH have been demonstrated by other studies showing that NOTCH also plays a role in inflammation, including in the JAK/STAT signaling pathway noncanonically. In breast cancer, the activation of NOTCH upregulated IL-6 expression via NOTCH ICD which then led to the activation of JAK/STAT signaling [99]. Meanwhile, another study showed that the increased expression of IL-6 in trastuzumab-resistant human gastric cancer (NCI-N87-R) cell line induced the expression of JAGGED 1, which then in turn further promoted the expression of IL-6. The increase in IL-6 then causes the activation of STAT3, suggesting that both the NOTCH/JAGGED 1 and IL-6/STAT3 pathways synergistically worked together in a positive feedback loop. To support that data, the suppression of the NOTCH pathway with *γ*-secretase inhibitor was then later showed to be able to reduce the IL-6 expression in NCI-N87-R cells [100].

In 2012, a study showed that UVB increases IL-6 mRNA and protein expression along with STAT3 phosphorylation at Tyr705 and complement factor B (CFB) mRNA 24 h after irradiation [101]. To determine the pathway induced by UVB, inhibition of JAK2 with JAK2 inhibitor AG490 or silencing of STAT3 expression was found to inhibit the expression of CFB mRNA. Treatment with tannic acid also causes the inhibition of STAT3 phosphorylation and CFB mRNA expression by inhibition of IL-6 protein, confirming that the IL-6/JAK/STAT signaling pathway was involved in UVB-irradiated RPE cells [101]. Despite not fully understanding the implications of the activation of these pathways, the increase of STAT3 protein may bode the start of AMD. In the globes of patients with wet AMD, the increase of phosphorylated STAT3 was detected and restricted to the RPE cells found in the areas of developing scars. This is an indication that during the proliferative stage of wet AMD, the RPE cells were highly activated and that STAT3 is potentially involved in the development of choroidal neovascularization [102]. Although the full mechanism of UVB damage on RPE and the relationship between the NOTCH and JAK/STAT pathways in RPE are not fully elucidated, the upregulation of both pathways by UVB might indicate the involvement of both pathways as illustrated in Figure 3. Further studies involving these pathways should be done to help better understand and prevent UVB-induced damage of RPE cells, particularly given that oxidative stress and inflammatory changes are known to be part of the underlying pathophysiology of AMD.

### 3.5. MAPK/P13K-AKT/p53 Mediated Cell Cycle Arrest and Cell Death

Many *in vitro* studies have shown that RPE cells which were irradiated with UVB had displayed increased cell death, DNA fragmentation, and apoptosis [98, 103, 104]. Microscopic images of microvillus shedding, nucleolus degeneration, the formation of vesicular structures, and mitochondrial degradation are the hallmarks of apoptosis that were seen in ARPE-19 cells after UVB irradiation [104]. Cell cycle arrest at the S phase was also reported by Chou et al. [105] when ARPE-19 cells were irradiated by UVB at doses of 10 mJ/cm^2^ and above. Cyclin E was also found to be upregulated while cyclin B and D were downregulated after irradiation. As cyclin E is responsible for the transition of cells from the G1 phase into the S phase while cyclins B and D are involved in the M phase and G1 phase, respectively, it is possible that with the accumulation of cyclin E, the cells were able to enter into the S phase but unable to proceed with mitosis due to the decrease in cyclin B [105–107].

On another note, when irradiated with 10-40 mJ/cm^2^ UVB, the phosphorylated ERK1/2 and AKT were dose-dependently increased after 24 hours. The phosphorylation of p53 was also significantly increased at 24 hours post-UVB irradiation, while the protein expression levels of SIRT1 were decreased [105]. To understand the involvement of ERK, AKT, and p53 in UVB-induced cell death, the cells were pretreated with three inhibitors targeting P13K, MEK/ERK, and p53 and an activator targeting SIRT1 for 1 hour before irradiation by Chou et al. [105]. The results yielded an increase in cell viability when the cells were treated with MEK/ERK inhibitor and SIRT1 activator. The increase in SIRT1 expression levels also significantly decreased phosphorylated ERK1/2 expression levels but only slightly decreased the expression of phosphorylated AKT. This indicates that SIRT1 is involved in the survival of the cells, and the suppression of this protein by UVB may result in cell death via the ERK pathway. SIRT1 activator also decreased the expression of cyclin E as previously mentioned and is suggested to be involved in cell cycle arrest at the S phase. This data correlates with the increase in cell viability after the increment of SIRT1 expression. Other than that, the inhibition of P13K yielded a decrease in the phosphorylation of AKT and ERK. Cyclin E and phosphorylation of p53 were also decreased by P13K inhibitor, suggesting an involvement of the AKT pathway in cell cycle arrest as well, although the inhibition of AKT alone was not enough to increase the number of viable cells. Lastly, treatment with p53 inhibitor did not change to cyclins B, D, and E, suggesting that there might be multiple pathways involved in UVB-induced cell cycle arrest. Upregulation of phosphorylated p38 was also seen after UVB irradiation on ARPE-19 cells, but more studies still need to be done to determine the role of p38 in the apoptosis process of the cells [108].

**Figure 4:**
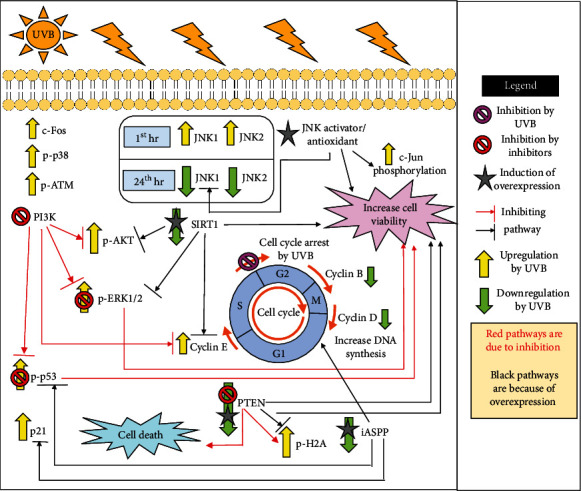
An overview of UVB-induced MAPK/P13K-AKT/p53-mediated cell cycle arrest and cell death in ARPE-19 cells. The cells were irradiated with UVB, and the changes in protein expression in each pathway illustrated were studied using inhibitors, activators, and antioxidants.

Besides that, studies have also addressed the involvement of p53 in UVB-induced cell death [33, 109, 110]. After irradiating the ARPE-19 cells with 30-90 mJ/cm^2^ UVB, the degree of elevation of phosphorylated p53 was obtained [110]. Likewise, the p53 mRNA level was also reported to increase almost 5-fold higher than the unexposed control in DrRPE cells [33]. In the study by He et al. [109], they also measured the expression of phosphorylated ataxia telangiectasia mutated (p-ATM), phosphorylated histone H2A (p-H2A), and phosphatase and tensin homolog (PTEN). Both p-ATM and p-H2A were found to be upregulated by UVB while PTEN was negatively regulated. He et al. [109] then proceeded to overexpress PTEN which resulted in a decrease in p-H2A and an increase in cell viability. On the other hand, when PTEN was silenced, not only was p-H2A increased but even the phosphorylation of p53 was increased together with cell apoptosis. Treatment with p53 inhibitor, however, was able to decrease the apoptosis of ARPE-19 cells even with PTEN was silenced, indicating a positive involvement of p53 with UVB-induced cell death. Research by Yan et al. [110] showed not only an increase in p53 but also an increase in p21 and a decrease in the inhibitor of apoptosis-stimulating p53 protein (iASPP) protein expression. The DNA synthesis of the ARPE-19 cells was also decreased after UVB exposure. When iASPP protein was induced to overexpress, the cell viability and DNA synthesis were increased, whereas p53, p21, and apoptotic cells were decreased. Hence, it can be said that iASPP plays a role in the regulation of p53, p21, and the survival rate of the UVB-damaged RPE cells.

c-Jun NH_2_-terminal kinase (JNK) or also known as stress-activated protein kinase is mainly activated under the duress of various stressful conditions. One of the activators of the JNK pathway in the ARPE-19 cells is the irradiation of UVB. In two separate studies, JNK was reported to be increased and subsequently decreased by UVB. Silván et al. [108] reported that the phosphorylation of JNK1 and JNK2 were increased in the ARPE-19 cells within an hour of irradiation. Another study had also shown that both the gene and protein levels of c-Fos were increased within 2 hours of irradiation [111]. On the other hand, after 24 hours of incubation following irradiation, the phosphorylation of JNK1 and c-Jun displayed significantly decreased levels as compared to nonirradiated controls. When ARPE-19 cells were pretreated with EGCG, a well-known antioxidant, or with anisomycin, a JNK activator, the phosphorylation level of JNK1 was increased together with the percentage of viable cells 24 hours after irradiation. EGCG was also able to decrease ROS production and increase the phosphorylation of c-Jun as well [51]. This shows that JNK has a complicated role in UVB-induced cell death, and it seems possible that changes in its expression may have a role in attenuating UVB-induced cell death. Additionally, it appears that antioxidants may be able to somewhat attenuate the damage caused by UVB which may help to reduce the damage to RPE cells and possibly reduce the incidence of AMD.  Figure 4 summarizes the different pathways in RPE cells that were activated by UVB irradiation, leading to cell death.

### 3.6. UVB Activates Intrinsic Apoptosis in RPE Cells

The cell apoptosis of RPE by UVB was via the intrinsic apoptosis pathway. After irradiating ARPE-19 cells with UVB, the gene expression of proapoptotic BCL-2-associated X protein (BAX) was upregulated while the mRNA expression of antiapoptotic B-cell lymphoma 2 (BCL-2) was downregulated [112]. Besides that, the upregulation of cytochrome C protein was also recorded by Balaiya et al. [112], which suggests that the integrity of mitochondrial membranes was compromised. This data agrees with other studies that reported the loss of mitochondrial potential and the number of mitochondria in ARPE-19 and DrRPE cells following exposure to UVB irradiation [33, 103, 113]. Another interesting study displayed vast changes in the mitochondrial morphology and movement before and after UVB irradiation in ARPE-19 cells. Before irradiation, it could be seen that within the cells, there was a bidirectional movement of short and elongated mitochondria with dense threads radially arranged around the nuclei or loosely arranged throughout the cytoplasm. Movement branches were also noted in many mitochondria. However, immediately after irradiation, the morphology of mitochondria was shortened and no longer moving with very few branched mitochondria remaining within the cells [114]. Comparable conditions were also reported by Youn et al. [45], whereby fewer, shorter, fragmented, and merged mitochondria were seen after UVB exposure.

**Figure 5:**
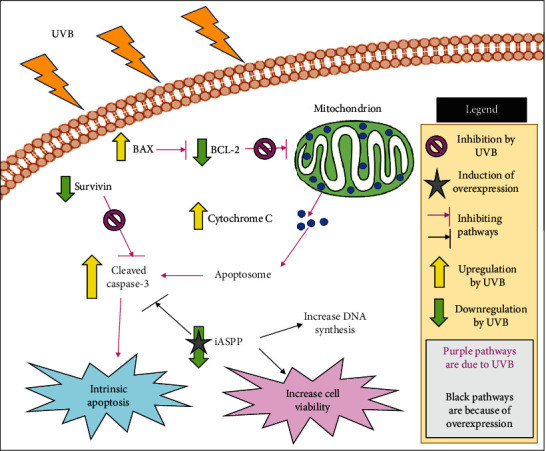
Irradiation of UVB on RPE cells activates intrinsic apoptosis in RPE cells by upregulation of proapoptotic proteins and downregulation of antiapoptotic proteins.

Other than the changes in BAX, BCL-2, and cytochrome C, the expression of cleaved caspase-3 was also increased following UVB irradiation in RPE cells [33, 110]. The increase in cleaved caspase-3 was suggested to be due to the decrease in iASPP protein expression. Yan et al. [110] had shown that when ARPE-19 cells were irradiated, the cells experienced a decrease in iASPP expression. However, when induced to overexpress iASPP protein and subjected to UVB irradiation, the percentage of cell viability and DNA synthesis were increased, while the percent of cell apoptosis and expression of cleaved caspase-3 were decreased. Yet, despite the changes in cleaved caspase-3, there were no changes to be seen in the expression levels of pro-caspase-3 throughout the experiment. The expression of survivin mRNA was also found to be decreased in a study on UVB-irradiated D407 cells [104]. In the study of melanoma cancer, it had been found that survivin, an antiapoptotic protein from the IAP family, is able to protect the cells from apoptosis [115]. Other studies have also shown that survivin is an inhibitor of activated caspase-3 and caspase-7 [116, 117]. Hence, with the decrease in survivin expression, it can be suggested that UVB mediates cell apoptosis and cell death via the intrinsic apoptosis pathway. An illustration of UVB irradiation activating intrinsic apoptosis in RPE cells can be seen in Figure 5.

### 3.7. Effect of UVB on Phagocytosis Activity in RPE

Phagocytosis is one of the most essential functions of RPE as it aids in the maintenance and support of photoreceptors. In our retina, photoreceptors are constantly synthesizing and forming new outer segment disks at a very high rate, slowly elongating its outer segments. Phagocytosis and degradation of the distal tips of the outer segments of the rods and cones, a process known as disk shedding, help compensate for the increased length of photoreceptors [118]. The loss of phagocytotic activity is one of the many etiologies of AMD. When studying RPE from AMD donors (age range 65-88) and normal control donors (age range 61-79), it was noted that there was a significant and dramatic decrease in phagocytosis in AMD donor eyes as compared to the control [119]. Moreover, Murad et al. [120] detected a significant inhibition in the expression of microRNA 184 (miR-184) in the primary RPE cultures of AMD in comparison to normal donor eyes. They discovered that the inhibition of this microRNA causes downregulation of ezrin expression and in turn decreases the expression of lysosomal-associated membrane protein-1 (LAMP-1) which affects the phagocytotic activity in RPE [84]. To determine if the irradiation with UVB can affect the phagocytotic activity of RPE cells, Youn et al. [113] proceeded to irradiate ARPE-19 cells with 50-200 mJ/cm^2^ UVB. As discovered, UVB exposure does induce a dose-dependent decrease in phagocytotic activity in RPE cells. The same decrease in phagocytotic activity was also reported by Youn et al. [45] after the application of 0.2 and 0.4 J/cm^2^ UVB on ARPE-19, while DrRPE cells displayed suppressed phagocytotic activity after exposing the cells to 4.2 J/cm^2^ UVB [33]. This is cause for concern as the renewal of the photoreceptor outer segments (POS) is key in maintaining vision. Thus, this warrants a more in-depth molecular and mechanism study on the effect of UVB on the phagocytotic role of RPE.

### 3.8. Increased Autophagy in RPE Cells as a Protection against UVB Oxidative Stress

Other than phagocytosis, the autophagic activity of RPE after irradiation was studied as well. Autophagy is a catabolic process where the cells degrade and recycle molecular material and organelles in cytoplasmic vesicles known as autophagosomes, with the aid of lysosomes [121]. This process not only removes unwanted biomaterials within the cells but also acts in response to environmental stress, prolonging viability [122]. In previous studies, dysregulation of autophagy had been associated with the pathogenesis of AMD and retinal damage. In 2009, a study had discovered that the drusen formed within the eyes of older human donors had increased markers for autophagy. It was also suggested that there is a positive correlation between the increase in the expression of autophagic markers and increased damage to mitochondrial DNA [123]. Another study had found that acute oxidative stress brought about an increase in autophagy in RPE while autophagy was decreased under chronic oxidative stress. Suppression of autophagy activity leads to increased generation of ROS, activation of the NLRP3 inflammasome, exacerbated reduction of mitochondrial activity, decreased cell viability, and most importantly increased production of lipofuscin [81, 124]. Hence, autophagy is essential in the removal of lipofuscin, and failure to remove it can lead to AMD [63, 125].

According to *in vivo* studies on human donor eyes, autophagy flux was initially increased at the early stages of AMD but significantly reduced as it enters the later stages. However, regardless of the stage of AMD, the autophagosome fractional volumes were substantially reduced as compared to age-matched controls, suggesting an impairment of autophagy in RPE cells of AMD patients [124]. According to Mitter et al. [124], the initial increment of autophagic flux was deemed to act as protection against oxidative stress, but as prolonged oxidative stress was induced within the cells, the ability of the autophagic system plateaus and is thus unable to efficiently remove the increasing number of damaged intracellular organelles within the cells which then accumulate triggering pathology.

The conversion of the microtubule-associated protein 1 light chain 3 (LC3) to the cytosolic form of LC3 (LC3-I) and then to LC3-phosphatidylethanolamine conjugate (LC3-II) occurs during the formation of autophagosomal membranes and is then localized onto autophagosomes and autophysosomes, which are the fusion of autophagosome and lysosomes [126]. Therefore, the increase in LC3-II is used as a marker in determining the formation of autophagosomes in RPE cells. As UVB irradiation is one of the causes of oxidative stress in RPE cells, the autophagic activity in RPE cells was measured and a significant increase in the LC3-II levels in ARPE-19 cells was seen. This data was also supported by microscopic images of the cells that showed an increase in the number of double-membrane vacuoles which is the typical appearance of autophagosomes [127]. Overall, this suggests that due to the damage induced by UVB exposure, the autophagic activity was activated in the RPE cells. However, the effect of long-term exposure and damage on autophagic activity had yet to be studied.

Besides measuring the conversion of LC3-II, changes in the mammalian target of rapamycin (mTOR) signaling pathway had also been investigated. mTOR is a serine/threonine kinase that is highly conserved and has a significant role in regulating cell metabolism and growth [128]. mTOR has two different signaling complexes known as mTOR complex 1 (mTORC1) and mTOR complex 2 (mTORC2). Of the two complexes, mTORC1's role is to regulate cell growth and cell proliferation by promoting anabolic cellular metabolism and inhibit catabolic processes like autophagy [129]. During oxidative and nitrosative stress, negative regulation of the mTOR pathway was found to induce autophagy [130, 131]. In the study done by Li et al. [127], the phosphorylation of two downstream markers of the mTOR pathway, ribosomal protein S6 kinase (S6K) and eukaryotic translation initiation factor 4E-binding protein 1 (4E-BP1), were downregulated after UVB irradiation. Both of these proteins are major substrates in the mTORC1 pathway and are involved in the regulation of protein synthesis [129]. With the downregulation of both these substrates, this indicates that the mTOR pathway was repressed under UVB-induced oxidative stress, supporting the previously mentioned increase in autophagy activity after UVB irradiation.

### 3.9. Osmoregulation and Water Permeability

The inner and outer BRB are formed from the tight junctions of retinal capillaries' endothelial cells and RPE cells, respectively [132]. Its key function is as a physiological barrier that is particularly restrictive towards the flux of protein, ions, and water in and out of the retina [133]. To facilitate the transport of water across the RPE, the cells use aquaporins. Aquaporins (AQP) are small, hydrophobic integral membrane proteins, and aquaporins 0-12 had been found in native RPE cells, with each having its functions [134, 135]. Interestingly, a study had shown that UVB was able to affect the water permeability and expression of AQP1, which is involved in maintaining the drainage and secretion of aqueous humor, in ARPE-19 cells [135, 136]. AQP1 in RPE *in vivo* is thought to contribute to efficient transepithelial water transport which if impaired may lead to subretinal edema, which may be linked to AMD. When irradiated with 20 mJ/cm^2^ UVB, the water permeability and the protein expression of AQP1 in the cells were found to be downregulated. UVB exposure also resulted in a marked increase in the generation of ROS and phosphorylation of ERK proteins. To determine if the induction of ROS and the activation of the ERK pathway are involved in the decrease of AQP1, ROS production was inhibited by N-acetyl cysteine (NAC), a general antioxidant, while the phosphorylation of ERK was inhibited using MEK/ERK inhibitors separately. The results obtained showed an increase in AQP1 protein expression, confirming that both the ROS and ERK pathways are involved in the suppression of AQP1 expression. In turn, an increase in the expression of AQP1 returns the water permeability of the cells to normal [136]. This suggests that UVB is able to induce damage to the eyes via the disruption of water permeability in RPE cells by activation of the MEK/ERK pathway.

**Figure 6:**
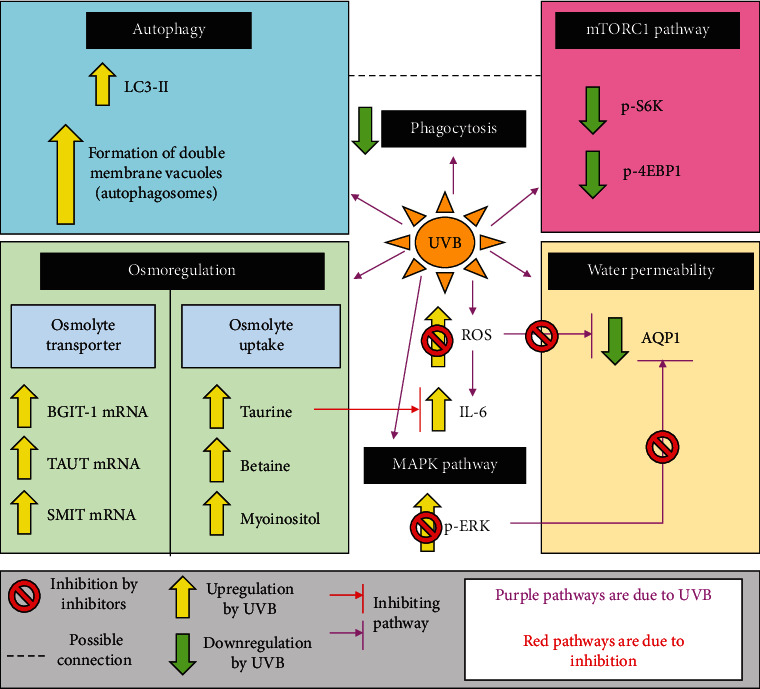
Essential functions of RPE such as autophagy, osmoregulation, phagocytosis, and its control on water permeability are affected by UVB irradiation.

Besides water permeability through the BRB, osmoregulation is essential in preserving cell health as well. Osmoregulation of the cells with organic osmolytes such as taurine, betaine, and myoinositol helps to keep the RPE cell shape during volume perturbations [137, 138]. Of all the osmolytes, taurine had been found to play a key role in the maintenance of retinal structures and functions. It is also the most abundant amino acid in the retina and is the most highly concentrated in the photoreceptor cells and RPE. This is due to the active uptake of the amino acid via sodium-dependent cotransporters from choroidal blood [139]. Functions such as antioxidant defense, stress responses, stabilization of proteins, and osmoregulation involving the regulation of cell volume homeostasis are among its many functions in the retina [140]. In 2015, it had been found that irradiation of RPE with UVB induces an increase in osmolyte transporter mRNA expression, which in turn increased osmolyte uptake. Dayang and Jinsong [141] showed that ARPE-19 cells were irradiated by UVB at 30 mJ/cm^2^, and betaine/GABA transporter (BGIT-1) mRNA and taurine transporter (TAUT) mRNA experience a significant increase in expression up to 700 and 383% as compared to cells in normatonic conditions 24 hours after irradiation. Sodium/myoinositol (SMIT) mRNA also experienced a slight increment after UVB irradiation. The cells also displayed a significant increase in the uptake of the osmolytes betaine, myoinositol, and taurine. Irradiation of UVB also upregulated IL-6 which is a proinflammatory cytokine. However, the uptake of taurine was able to suppress the increase in IL-6 expression. Hence, the increase in osmolyte uptake after UVB irradiation was suggested to be a protective measure against UVB damage especially taurine. An overview of the effect of UVB on autophagy, phagocytosis, osmoregulation, and water permeability of RPE cells has been illustrated in Figure 6.

On another note, it is essential to maintain ion channels, such as potassium, chloride, calcium, sodium, and zinc ion channels, in RPE cells as dysregulation can potentially lead to AMD [142, 143]. An example of dysregulation of zinc ions was described in the retina of cynomolgus monkeys with early-onset macular degeneration. In the retina of these monkeys, a fourfold decrease in zinc was observed [144], while another reported a significant increase in zinc levels in the maculae and sub-RPE deposits, which include basal laminar deposits and drusen, in AMD patient eyes [145]. Hence, for future studies, it would pose to be interesting to see the effect of UVB on these channels.

## 4. Bioactive Compounds from Natural Products against UV Damage on RPE

Through the ages, a significant number of potential bioactive compounds are from natural sources and these have greatly contributed to many different fields including medicine, cosmetic studies, and agriculture [146–151]. As can be seen in Table 2, some of the bioactive compounds reported by various studies are antioxidants and thus are able to inhibit the increase in ROS generated by UVB. While the total number of studies focusing on RPE is limited, the data available is already very promising. By decreasing the ROS levels in the cells, this reduces the oxidative stress in RPE and therefore leads to increased cell viability and decreased cell apoptosis [52, 103]. Some of the compounds studied are also found to be actively accumulated within the macular region of the eye, for example, carotenoids like lutein and zeaxanthin which are known as macular pigment [152]. These accumulations of macular pigments are easily obtained from fruits, vegetables, and eggs and have been reported to have photoprotective properties against light-induced damage [153]. Other antioxidants like epigallocatechin gallate from green tea had shown photoprotective properties against UV light on the skin [154].

Although more connections and cross-talks between the different pathways had yet to be elucidated, it is undeniable that natural products hold vast potential in the prevention against UVB damage to RPE. In light of that, Table 3 further tabulates a few additional examples of bioactive compounds from natural products that were able to attenuate the effects of UVR, white light, or chemically oxidative stress on RPE or the retina. From these research studies, it can be seen that for future studies, not only is there more bioactive compounds to explore but there are more pathways to investigate. For example, the Nrf2 antioxidant pathway had been known to regulate cytoprotective responses whenever cells are under ROS- and electrophile-induced endogenous and exogenous stress [155]. Besides that, the effect of UVB on the vascular endothelial growth factor (VEGF) expression should also be investigated as the increase in VEGF is known to lead to choroidal neovascularization in AMD [156]. Additionally, more *in vivo* studies including bioactive compounds too need to be done as well.

## 5. UVB Induced Retinal and Potentially RPE Damage in *In Vivo* Models

To further demonstrate the detrimental effect of UVB damage, *in vivo* studies were conducted as well. However, the difference between *in vitro* and *in vivo* studies is that while *in vitro* studies focused on RPE alone, *in vivo* studies focused on changes in the whole retina. As of now, there is no known *in vivo* study on the effects of UVB on RPE alone. However, based on the current studies, one can still obtain a holistic view of the changes UVB does to the retina and potentially towards the RPE. For example, a study was conducted on the eyes of Wistar albino rats with AMD. In this experiment, the eyes of these rats were laid open and irradiated with either 500 mJ/cm^2^ of UVA or 35 mJ/cm^2^ of UVB. After that, any damage to the eye structure and the changes in retinal proteome were analyzed [168]. Firstly, it is important to note the ~14-fold difference in irradiation dosage between UVA and UVB. This difference in the energy level highlights the phototoxicity of UVB in comparison to UVA, and from the results, it became even evident that the detrimental effects of UVB were much severe than UVA on the retina. Although both groups of rats displayed classical symptoms of retinal degeneration like neovascularization, increased accumulation of drusen, and depigmentation, 495 protein expressions were attenuated by UVB while UVA only modified the expression of 53 proteins in the retina. In total, 45 proteins were found to overlap between UVA and UVB irradiation [168]. Similarly, in another study on mice, several genes too experienced significant modifications in their expression after UVB irradiation. In this research, the retina of 10-week-old mice was exposed to 35 mJ/cm^2^ UVB once daily for 4 consecutive days. After 2 days of recovery, the mice were sacrificed and the effect of UVB on the retina was studied [169]. Structurally, no changes were seen in the retinal structures after irradiation, and yet, 126 genes were significantly upregulated while 51 other genes were downregulated in the retinal tissue. After analysis, these genes can be sorted into 5 categories of genes and the categories were chromatin regulation, stress and signaling, gene expression regulation, RNA processing, and neuronal genes [169]. Based on these categories, it can be seen that UVB does affect gene expression down to the chromosome level and induces cellular stress upon irradiation. When comparing the changes in protein expression, several oxidative stress proteins such as glutathione-S-transferase, SOD, CAT, and peroxiredoxin-2 were increased, while aldehyde dehydrogenase was decreased [168]. As reported previously, changes in SOD and CAT expression levels too were observed in the ARPE-19 cell line when the cells were irradiated [52]. This confirms that UVB does affect the endogenous antioxidant defense system on both RPE and the retina as a whole.

Of all the proteins that were upregulated or downregulated, the significant downregulation in lumican after the UVB irradiation poses to be an interesting point to look into [168]. This is because, previously, the secretion of lumican by RPE cells was reported to be affected in AMD patients, but the expression levels in AMD are contradictory to the ones seen after UVB exposure [170]. It was reported instead that lumican was upregulated and secreted 2-fold higher by AMD human donor RPE cells, with the Y402H-complement factor H variant, as compared to normal human RPE cells [170]. Lumican is a keratin sulfate-carrying member of the small leucine-rich proteoglycan family (SLRP) that had been known to be essential in the formation of the cornea during the development of the embryo and continues to maintain corneal topography in adults [171, 172]. Mutant mice that were deficit in lumican were reported to have fragile skin, due to loose and disorganized arrangement of dermal connective tissue, and corneal opacification that were increasing with age [173]. Although the decrease in lumican expression is often related to changes in the transparency of the cornea in ocular studies, Bonilha et al. [174] suggest that the protein may play a part in the attachment of the retina to RPE cells as they found expressions of lumican and fibromodulin in mouse RPE microvilli. Another study using lumican, fibromodulin, and lumican-fibromodulin knockout mice reported that the sclera was thinner in all models, but the double-knockout mice even experienced retinal detachment. Further analysis then showed that in lumican or fibromodulin knockout mice, the expression of the lumican and fibromodulin was significantly increased, respectively, as though in compensation of the knockout protein [175]. Despite the double-knockout mice displaying retinal detachment and the contradictory results in lumican expression from AMD patients, it is still undeniable that lumican functions together with fibromodulin in the role of retinal attachment and any changes to its expression level could lead to irreversible vision loss. However, there is a possibility that this contradictory result could be due to UVB exacerbating the degeneration of the AMD eyes by instigating a high number of RPE cell death, leading to a sudden decrease in lumican expression as compared to the unexposed control.

In a nutshell, although UVB may not change the morphology and structure of the retina, both studies had proven that UVB still does affect the transcription of genes within the retina. Despite that, both studies have not yet accounted for the effects of prolonged exposure of UVB on the retina and thus how accumulative damage from UVB may do to the retina, leading to AMD. Therefore, not only studies on the genes mentioned should be done but also investigations on the effect of UVB on the retina as a whole and RPE as an individual cell type during long-term exposure have to be conducted as well.

## 6. Conclusion

In conclusion, UVB could cause damage to the RPE cells via direct DNA damage, oxidative stress, and activation of several different pathways, such as NLRP3, MAPK, P13K-AKT, NOTCH, and JAK/STAT, which leads to inflammation, cell apoptosis, and cell death. Besides decreasing cell viability, exposure to UVB also affects phagocytosis, osmoregulation, and water permeability of RPE cells. To combat the degenerative effect of UVB, melanin within the RPE was seen to offer photoprotection to RPE, but this protective effect slowly disappears with time as melanin degrades with age. However, the increase in the expression of LEDGF and autophagic activity could aid as protection against UVB-induced oxidative stress. Other potential treatment against AMD is the bioactive compounds from natural products. These compounds were able to attenuate ROS production and thus reduce the detrimental effects of UVB on RPE, making them suitable therapeutic candidates. On another note, despite current *in vivo* studies being on the whole retina, the effects of UVB on the retina showed changes in retinal morphology and aberrations in gene and protein expressions, pointing towards cellular stress, depigmentation of the retina, increased drusen formation, mutagenic changes in the chromosome level, and possibly retinal detachment. Hence, based on what was discovered, more studies on both *in vitro* and *in vivo* studies ought to be done to fully understand the mechanisms and pathways behind UVB-induced RPE cell damage. Other than that, further investigation of other naturally available bioactive compounds should be pursued as well for more potential treatments against UVB-induced macular degeneration.

## Figures and Tables

**Table 1:** Characteristics and drawbacks of various established and primary RPE cell lines.

Model	Species	Type of cell line	Source	General characteristics and drawbacks	References
ARPE-19	Human	Established	Globes of a 19-year-old male donor who had passed away 2 h before extraction	*General characteristics*: (i) “Cobblestone appearance” and forms a monolayer (ii) Increasing pigmentation in cells with passages (iii) Formation of domes, indicating ability to pump ions vectorially (iv) Expresses cellular retinaldehyde-binding protein (CRALBP) and RPE-specific protein (RPE65), which are components in the retinoid visual cycle (v) Diploid karyology *Drawbacks*: (i) Low transepithelial resistance (TER) measurement which could compromise barrier functions (ii) Unable to obtain complete polarization (iii) Heterogenous expression of CRALBP (iv) Varied in pigmentation across different cells (v) Inconsistent formation of microvilli (vi) Deletion in chromosome 8 and addition in chromosome 19 (vii) Features such as morphology, retinoid metabolism, secretion of vascular endothelial growth factor (VEGF), and pigment epithelium-derived factor (PDEF) become compromised with increasing passages	[24, 40, 41]

D407	Human	Established	Globes of a 12-year-old white male	*General characteristics*: (i) “Cobblestone appearance” and forms a monolayer (ii) Has intercellular junctional complexes linking tightly apposed membranes by N-cadherin (iii) Has cortical microfilaments parallel to the plasma membrane and actin filaments bound to the lateral surface of the cells (iv) Short microvilli on apical surfaces of the cells (v) Contained keratins 7, 8, 18, and 19 and formed the keratin filament network throughout the cytoplasm in confluent cells (vi) Expresses CRALBP, indicating the transport of retinoids (vii) Expresses vimentin, which is commonly expressed in many epithelial cells (viii) Able to subculture for more than 200 times *Drawbacks*: (i) Lack of pigmentation (ii) Form triploid karyotype when cells are more than 50 passages (iii) Low TER measurement (iv) Did not localize tight junction protein zonula occludens-1 (ZO-1) to cell borders, indicating impairment in barrier function (v) Loss of polarization in spectrin associated with the actin and transmembrane proteins (vi) Only one out of the three enzymatic reactions in the metabolism of vitamin A, retinol dehydrogenase (RDH) activity was observed	[25, 42]

DrRPE	Zebrafish	Primary cell line	Eyes of 15 3-month-old adult zebrafish	*General characteristics*: (i) Cell diameter is 15-19 *μ*m (ii) Optimum growth condition is at 28°C with 15% FBS (iii) Doubling time is around 40 h (iv) Forms monolayer of majority mononucleate (v) Cells form pigment granules in 10 days (vi) Expresses RPE65 and cytokeratin 19 (vii) Cells were able to be subculture more than 75 times and were able to recover from a year of liquid nitrogen storage even at passage 75 (viii) High plating efficiency (ix) Diploid chromosomes, ranging from 38-53 chromosomes, with 50 chromosomes being the most common number (x) Able to convert all-*trans*-retinol to retinyl esters, producing retinosomes *Drawbacks*: (i) Some binucleate cells were found in culture (ii) Loss of melanin granules with increasing passages	[33]

Bovine RPE cells	Bovine	Primary cell line	Globes of freshly slaughtered bovine	*General characteristics*: (i) Polygonal or cuboidal shaped lightly pigmented cells with round nuclei (ii) Expresses vimentin, S100, and cytokeratin proteins (iii) Has a slower growth rate as compared to the iris pigment epithelium, iris melanocyte, and choroidal melanocyte	[43]

**Table 2:** The photoprotective effect of bioactive compounds or extracts obtained from natural products against UVB-induced damage on RPE cells.

Bioactive compounds or extracts of natural product	Source of natural product	Concentration, type, and duration of treatment	UV range and dosage	Subject of study	Photoprotective effect on RPE	Citations
Lutein	Silkworm (Bombyx mori), marigold, vegetable, and fruits	(1) *First study*: (i) Concentration of 50 *μ*M or 25 *μ*M in combination with trolox, (ii) 4 h pretreatment before irradiation (2) *Second study*: (i) Concentration of 5 *μ*M, 1 h pretreatment before irradiation	(1) *First study*: (i) UVB (40 mJ/cm^2^) (2) *Second study*: (i) UVB (50 mJ/cm^2^)	ARPE-19 cell line	(1) *First study*: (a) Increase cell viability (b) Decrease lipid peroxidation (c) Decrease ROS and caspase-3 protein levels (d) Decrease glutathione peroxidase activity (e) Modulate catalase activity opposing to UVB-induced changes (2) *Second study*: (a) Increase cell viability (b) Decrease ROS (c) Decrease phosphorylation of JNK1/2 and p38	[52, 108, 153]

Zeaxanthin	Fruits and vegetables	(1) *First study*: (i) Concentration of 50 *μ*M or 25 *μ*M in combination with trolox, 4 h pretreatment before irradiation (2) *Second study*: (i) Concentration of 5 *μ*M, 1 h pretreatment before irradiation	(1) *First study*: (i) UVB (40 mJ/cm^2^) (2) *Second study*: (i) UVB (50 mJ/cm^2^)	ARPE-19 cell line	(1) *First study*: (a) Increase cell viability (b) Decrease lipid peroxidation (c) Decrease ROS and caspase-3 protein levels (d) Decrease glutathione peroxidase activity (e) Modulate catalase activity opposing to UVB-induced changes (2) *Second study*: (a) Increase cell viability (b) Decrease ROS (c) Decrease phosphorylation of JNK1/2 and p38	[52, 108, 153]

EGCG	Green tea	(1) *First study*: (i) Concentration of 5 *μ*M, 2 h pretreatment before irradiation (2) *Second study*: (i) Concentration of 50 *μ*M, 3 h pretreatment before irradiation	(1) *First study*: (i) UVB (50 mJ/cm^2^) (2) *Second study*: (i) UVB (100 mJ/cm^2^)	ARPE-19 cell line	(1) *First study*: (a) Decrease cell apoptosis (b) Decrease ROS (c) Increase JNK and c-Jun phosphorylation (2) *Second study*: (a) Increase cell viability (b) Decrease in LC3-II proteins expression level (c) Increase phosphorylation of S6K and 4E-BP1	[51, 127]

Cyanidin-3-glucosidase	Fruits and vegetables	Concentration of 5 *μ*M, 1 h pretreatment before irradiation	UVB (50 mJ/cm^2^)	ARPE-19 cell line	(a) Increase cell viability (b) Decrease ROS (c) Decrease phosphorylation of JNK1/2 and p38	[108, 157]

Tannic acid	Tea, coffee beans	Concentration of 25 *μ*M, 1 h pretreatment before irradiation	UVB (10 mJ/cm^2^)	ARPE-19 cell line	(a) Decreases IL-6 protein expression levels (b) Decrease STAT3 phosphorylation on Tyr705 (c) Decrease CFB mRNA expression levels	[101, 158]

*Lycium barbarum* ethanolic and aqueous extract	*Lycium barbarum*	Concentration of 25 *μ*M and 50 *μ*M, 2 h pretreatment before irradiation	UVB (50 mJ/cm^2^)	ARPE-19 cell line	(a) Increase cell viability (b) Decrease ROS (c) Decrease cell apoptosis and DNA damage (d) Prevent the loss of mitochondrial membrane potential	[103]

Green tea polyphenols	Green tea	(1) *First study*: (i) Concentration of 70 and 140 mg/L, 2 h pretreatment before irradiation and 2 h posttreatment after irradiation (2) *Second study*: (i) Concentration of 70 and 140 mg/L, 2 h pretreatment before irradiation and 2 h posttreatment after irradiation	(1) *First study*: (i) UVB (720 mJ/cm^2^) (2) *Second study*: (i) UVB (720 mJ/cm^2^)	(1) *First study*: (i) D407 (2) *Second study*: (i) D407	(1) *First study*: (a) Increase cell viability (b) Decrease DNA fragmentation (c) Increase expression of survivin gene (2) *Second study*: (a) Decrease c-Fos mRNA and protein levels	[104, 111]

**Table 3:** Examples of bioactive compounds from natural products that have been shown to have a protective effect on RPE or retina against UVR, white light, or chemically induced oxidative stress.

Bioactive compounds of natural product	Source of natural product	Subject of study	Protective effect on RPE	Citations
3H-1,2-dithiole-3-thione	Cruciferous vegetables	(1) *In vitro studies*: (i) ARPE-19 and primary murine RPE cells from C57/B6 mice (2) *In vivo studies*: (i) Male BALB/c mice	(1) *In vitro studies* (a) Protection against UVB+UVA2: (i) Inhibits cell death (ii) Attenuated cell apoptosis, caspase-9 activation, and mitochondrial membrane potential reduction (iii) Inhibits ROS production (iv) Increases Nrf2 and HO-1 protein expression dose- and time-dependently. HO-1 (but not Nrf2) mRNA was also increased (v) Increase in Nrf2 phosphorylation and nuclear accumulation (vi) Increase in *γ*-glutamyl-cysteine ligase catalytic subunit (GCLC), *γ*-glutamyl-cysteine ligase modifying subunit (GCLM), and NAD(P)H:quinone oxidase-1 (NQO-1) (vii) Disruption of Nrf2-Keap1 association in the cytosol (viii) Activated AKT-mTORC1 signaling time- and dose-dependently by upregulating phosphorylation of AKT, S6, and 4E-BP1 (b) Protection against H_2_O_2_: (i) Inhibits H_2_O_2_-induced cell death, cell apoptosis, and ROS production (2) *In vivo studies of the retina exposed to white light* (i) Attenuated the decrease in a- and b-wave amplitudes on the electroretinography (ERG) (ii) Decrease in light-induced apoptosis of the outer nuclear layer of the retina	[159, 160]

Escin	Seed of horse chestnut	ARPE-19 and primary murine RPE cells from C57/B6 mice	(1) *In vitro studies* (a) Protection against H_2_O_2_: (i) Inhibit cell death induced by H_2_O_2_ dose-dependently (ii) Decrease H_2_O_2_-induced cell apoptosis and caspase-3 activity (iii) Decrease in H_2_O_2_-induced ROS levels and lipid peroxidation (iv) Activation of Nrf2-ARE genes by increasing HO-1, SRXN-1, and NQO-1 mRNA (v) Induction and accumulation of Nrf2 phosphorylation at serine 40. The nuclear level of Nrf2 was also increased (vi) Induce AKT activation by inducing Ser-473 and Thr-308 phosphorylation	[161]

Salvianolic acid A	Root of *Salvia miltiorrhiza*	ARPE-19 and primary murine RPE cells from aged male C57BL/6 mice	(1) *In vitro studies* (a) Protection against H_2_O_2_: (i) Dose-dependently increase cell viability despite H_2_O_2_ treatment (ii) Decrease in H_2_O_2_-induced cell apoptosis and caspase-3 cleavage (iii) Inhibit MAPK activation by inhibiting the phosphorylation of ERK1, p38, and JNK (iv) Restore mTORC1 activation by increasing the phosphorylation of p-S6 and p-4E-BP1 (v) Inhibit H_2_O_2_-induced phosphorylation of AMP-activated protein kinase (AMPK) and ACC (vi) Increase protein expression of Nrf2 and HO-1 (vii) Increase phosphorylation of AKT and S6, indicating the activation of the AKT/mTOR pathway	[162]

Fucoxanthin	*Laminaria japonica* (marine brown alga)	(1) *In vitro studies*: (i) ARPE-19 cell line (2) *In vivo studies*: (i) Rabbit	(1) *In vitro studies* (a) Before the introduction of white light to RPE cells (i) Inhibit VEGF overexpression (b) Protection against white light exposure (i) Significantly improve the phagocytotic activity of RPE (ii) Inhibit RPE cell senescence (iii) Decrease ROS levels (2) *In vivo studies of the retina exposed to white light* (i) Prevent light-induced damage and improve microcirculation of the retina based on ERG datas	[163]

Resveratrol	Red grapes	ARPE-19	(1) *In vitro studies* (a) Protection against UVA (i) Increase survival rate of RPE cells (ii) Reduce the production of UVA-induced intracellular H_2_O_2_ (iii) Inhibit the induction of ERK1/2, JNK, and p38 (iv) Reduces the increase of COX-2 expression	[164]

Ginsenoside Rg-1	Ginseng	ARPE-19	(1) *In vitro studies* (a) Protection against CoCl_2_ (i) Increase the cell viability and inhibit cell apoptosis of RPE by decreasing caspase-3 activity (ii) Inhibit the production of ROS (iii) Inhibit the activation of JNK and p38 (iv) Activate mTORC1 by increasing the phosphorylation of S6 and 4E-BP1 (v) Increases the phosphorylation of AKT (vi) Inhibit the activation of AMPK (b) Protection against cell hypoxia (i) Restored cell viability and decreased cell apoptosis	[165]

Ginsenoside Rh3	Ginseng	(1) *In vitro studies*: (i) ARPE-19 (2) *In vivo studies*: (i) BALB/c male mice	(1) *In vitro studies* (a) Protection against UVB+UVA2 (i) Inhibit cell death caused by the exposure (ii) Attenuate cell apoptosis and cell necrosis (iii) Inhibits caspase-3 activity and histone-bound DNA accumulation (iv) Increases HO-1, NQO-1, and GCLC mRNA (v) Elevates Nrf2 protein level and translocation into the cell nuclei (vi) Attenuates ROS production (vii) Increases the expression of miR-141and decreases Keap1 mRNA, suggesting the activation of the Nrf2 pathway (2) *In vivo studies of the retina exposed to white light* (i) Increase both ERG a- and b-wave amplitude, protecting the retina against light-induced damage (ii) Increase of HO-1, NQO-1, and GCLC mRNA and protein levels in the retinal tissue (iii) Upregulation of Nrf2 protein in the retinal tissue (iv) Elevated miR-141 and decrease in Keap1 mRNA was detected	[166]

Luteolin	*Platycodon grandiflorum*	ARPE-19	(1) *In vitro studies* (a) Protection against T-butyl hydroxide (i) Attenuation of T-butyl hydroxide-induced cell death (ii) Reduced the ROS level (iii) Inhibit caspase-3 activation and reduced apoptotic nuclei and DNA fragmentation	[167]
